# Surgical treatment of avulsion fracture around joints of extremities using hook plate fixation

**DOI:** 10.1186/s12891-019-2585-1

**Published:** 2019-05-10

**Authors:** Qudong Yin, Yongjun Rui, Yongwei Wu, Jun Liu, Yunhong Ma, Sanjun Gu, Mingxia Zhou, Jiwen Yu

**Affiliations:** 1 Department of Orthopaedics, Wuxi No. 9 People’s Hospital Affiliated to Soochow University, No.999 Liangxi Rd, Wuxi, 214062 Jiangsu Province People’s Republic of China; 2 Department of Orthopaedics, Lianyungang People’s Hospital, Lianyungang, 222002 China

**Keywords:** Avulsion fractures, Hook plate, Internal fixation

## Abstract

**Purpose:**

This study proposed to access the clinical outcome of avulsion fractures around joints of extremities using the hook plate.

**Methods:**

A total of 60 patients with avulsion fractures of joints admitted in our hospital between January 2011 and June 2016 were performed the surgery of hook plate fixation. Functional recovery was evaluated using the Lysholm knee score, Kaikkonen ankle injury score, Mayo elbow and wrist function score, and Neer shoulder function score.

**Results:**

All the patients were healed within 3 months after surgery with stage I healing incision without vascular or nerve injuries. The average follow-up period was 18.1 months. At the last follow-up, no instability of joints, looseness of internal fixation or traumatic arthritis was observed. Mild joint fibrosis occurred in 5 cases. A total of 57 patients were well recovered with the excellent and good rate of 95%. Three patients with humeral avulsion fracture of the greater tuberosity had shoulder joint adhesion and peri humeral inflammation at the last follow-up due to the poor cooperation for early rehabilitation exercise. In the last follow-ups, the functional score of the affected limb was markedly greater than that in the 3-month follow-ups (*p* < 0.05).

**Conclusion:**

Hook plate fixation has the therapeutic effect on treating avulsion fractures around joints of extremities with the advantages of reliable fixation, early rehabilitation after operation, high recovery rates of joint function, wide indications, and convenient uses.

## Introduction

Joints are surrounded by articular capsule, ligaments and tendons. Avulsion fractures occur when the capsule, ligaments or tendons are avulsed from the bone due to unexpected contraction of the muscles or mechanical force [1]. As reported, the contractile properties of muscle and the attachment of tendons and ligaments at both ends of bone collectively contributed to the muscle contraction, so unexpected contraction of the muscles could lead to avulsion fractures at the site of tendon or ligament attachment [2]. Most avulsion fractures were intra-articular fractures, while relatively small fracture fragments are commonly attached to the ligament and capsule, making the joint difficult to return to the normal anatomical position [3]. The conservative treatment often has a poor therapeutic effect on avulsion fractures around joints, easily leading to fracture nonunion, joint instability and joint adhesion [4]. There are several fixations used to treat avulsion fractures, mainly including screw fixation [5], percutaneous Kirschner wire (K-wire) fixation [6], bone grafting [7], and plating fixation [8]. Internal fixation using lag screws or absorbable screws is recommended to fix large fragments, while K-wire and stainless-steel wire fixation is used to fix small fragments in order to avoid fracture nonunion [9]. However, the stability of screw fixation was not sufficient to meet the requirement of early rehabilitation exercise for patients with osteoporosis [10]. K-wire and stainless-steel wire fixation also shows unfavorable outcome, such as poor stability of the joint, which increases the risk of internal fixation failure.

Recently, accumulating evidence has strongly implied that the hook plate fixation has the advantages of more stable fixation of the fragments regardless of the bone quality and high accurate reduction in fracture with small fragments in the treatment of avulsion fractures [11]. For example, Shin et al. [12] found that the hook plate fixation provided stronger fixation relative to a suture anchor for thumb ulnar collateral ligament (UCL) fracture-avulsions. Moreover, Mehling et al. [13] have reported the excellent outcomes of mini-hook plate treating for phalangeal fractures. Based on these findings, we proposed that the hook plate might be a compatible fixation instrument for treating avulsion fractures around joints.

The purpose of the present study was to summarize clinical data of patients with avulsion fractures around joints of the extremities treated with hook plate fixation. More importantly, it was our goal to assess the necessity of surgery, the flaw of the mainstream internal fixation as well as the specific characteristics and therapeutic outcomes of hook plate.

## Material and methods

### Participants

A total of 60 patients who met the criteria were recruited for this study from January 2011 to June 2016. *Inclusion criteria*: (1) Avulsion fractures that were fit for the treatment of hook plate fixation, including the fractures of the humeral greater tuberosity, humeral condyle, ulna olecranon, ulnar styloid, ankle joint, and tibial condyle. (2) The width of the avulsed fracture bone is greater than 10 mm, which is larger than the opening of the hook plate. (3) The fracture separative shift shown in CT scan or X-ray is more than 5 mm. (4) Follow-up data were completed. *Exclusion criteria*: (1) Patients with concurrent severe fractures or vascular and nerve injury at the same joint, (2) The follow-up time was less than 12 months, (3) less than 18 years old.

There were 35 males and 25 females aged between 20 and 73 years (mean age 43.7). The causes of fracture included fall injury (*n* = 24), sports related injury (*n* = 17), falling injury (*n* = 11), and traffic injury (*n* = 8). Of the 60 patients, 19 cases were avulsion fractures of posterior cruciate ligament (PCL) at the tibial insertion, 6 cases were avulsion fracture of the humeral medial epicondyle, 8 cases were avulsion fractures of the humeral greater tuberosity, 6 cases were lateral malleolus avulsion fractures, 17 cases were ulna olecranon avulsion fractures, and 4 cases were ulnar styloid avulsion fractures. All fractures were fresh and unstable fractures. All patients presented to the clinic between 2 h to 19 days with the mean day of 6.6 days after injury. To confirm the diagnosis of avulsion fractures, patients received X-ray, computed tomography (CT) or/and Magnetic resonance imaging (MRI) examination. The general information of the patients was shown in Table 1.

**Table 1:** The general information of the patients

Fracture Site	Case	Gender (m/f)	Age	Hospital stays (days)	Preoperative Functional Score
Greater tuberosity of humerus	8	5/3	54.8 ± 19.1	6.9 ± 1.4	42.0 ± 2.1
Medial epicondyle of humerus	6	4/2	27.8 ± 7.7	6.0 ± 1.4	50.5 ± 1.9
Ulna olecranon	17	10/7	43.8 ± 13.8	7.6 ± 3.0	48.4 ± 1.9
Ulnar styloid	4	4/2	42.8 ± 12.7	5.5 ± 0.6	55.5 ± 2.1
Lateral malleolus of tibia	6	3/3	42.0 ± 14.2	6.7 ± 4.4	50.5 ± 2.1
Knee PCL	19	11/8	45.4 ± 14.5	6.4 ± 1.4	50.2 ± 1.4

*PCL* posterior cruciate ligament

### Operative technique

**Fig. 1:**
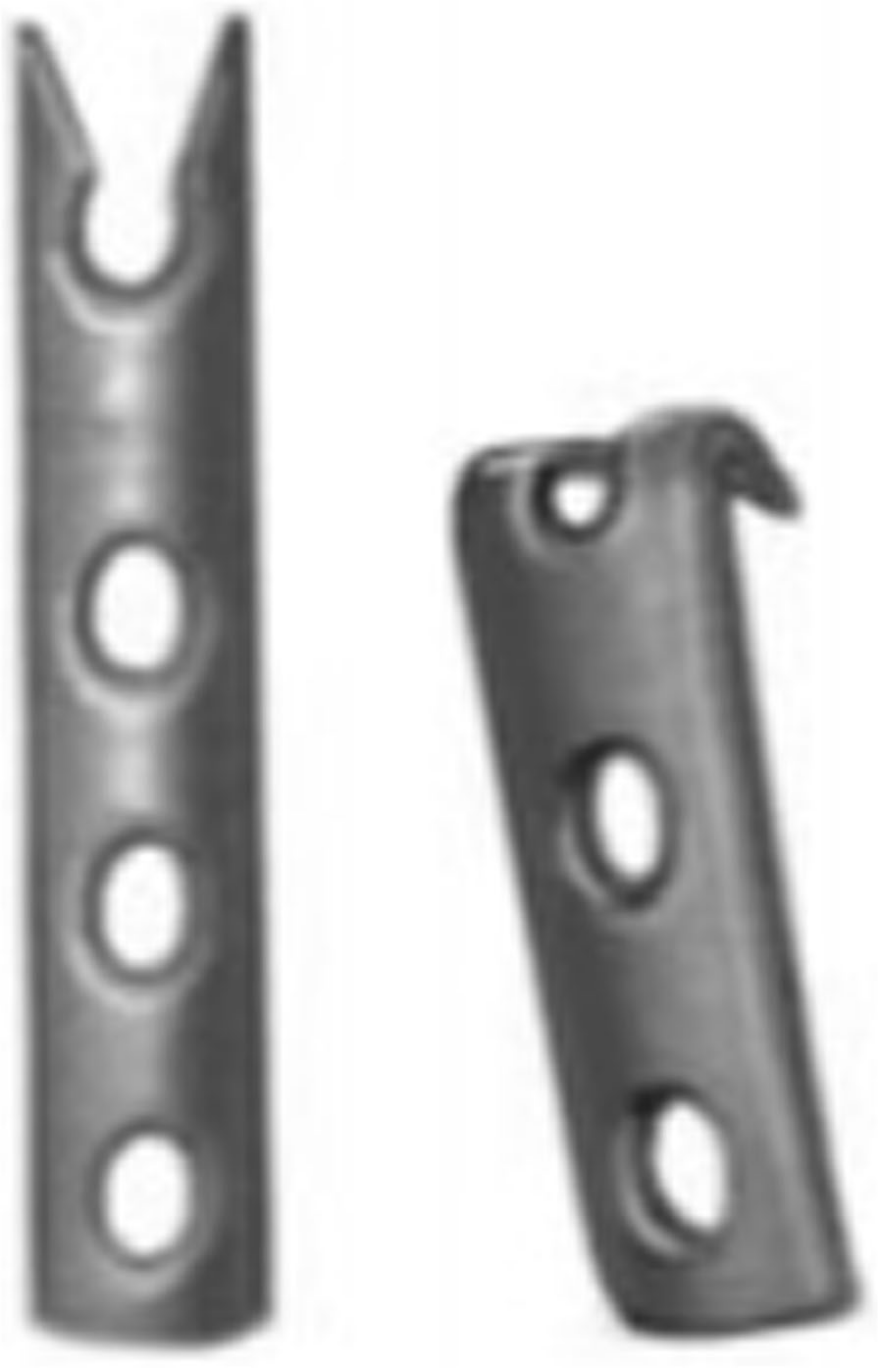
Diagram of hook plates with two symmetric sharp teeth hooks

**Fig. 2:**
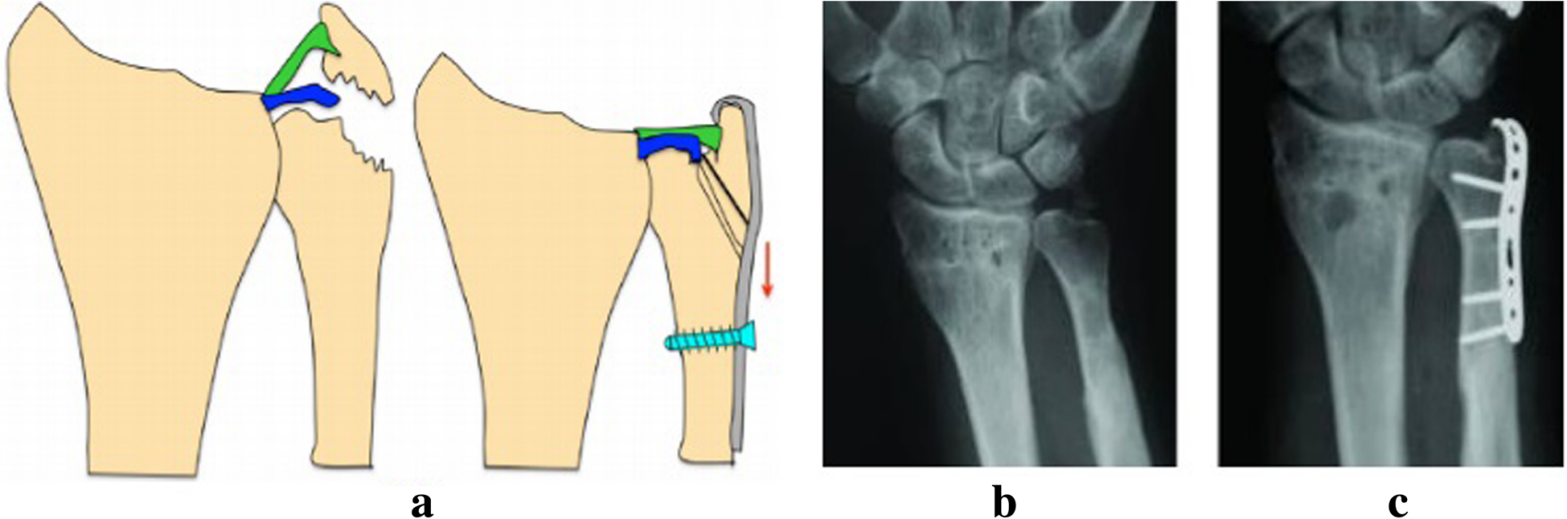
The radiographs of patients with ulnar styloid avulsion fracture treated with hook plate fixation. **a** The illustrations showing the hook plate procedure; (**b**) Preoperative posteroanterior X-ray films; (**c**) Lateral X-ray films after 3 months postoperatively

**Fig. 3:**
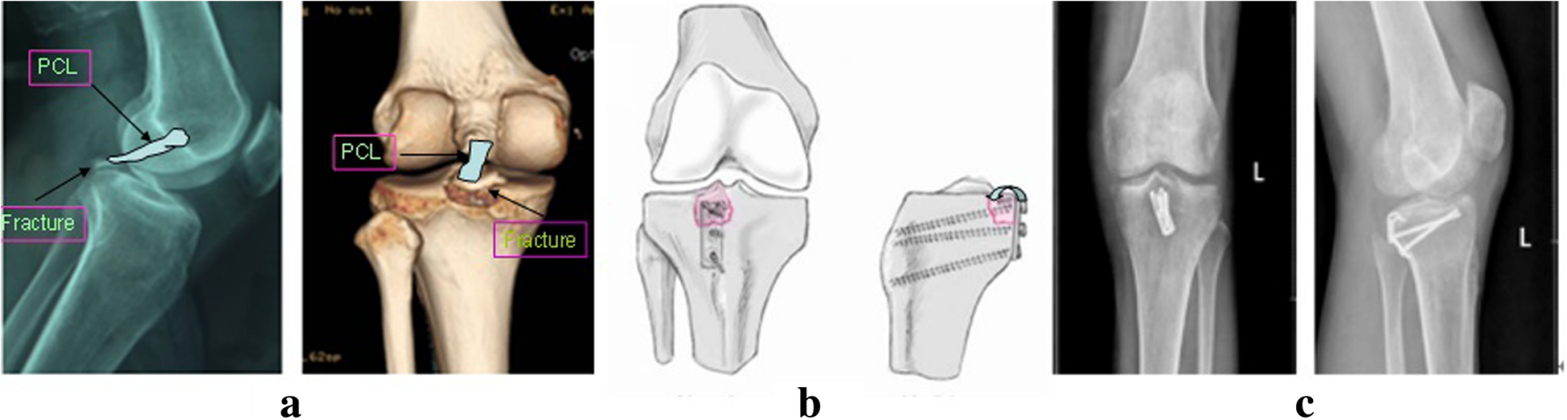
The radiographs of patients with avulsion fractures of PCL at the tibial insertion treated with hook plate fixation. **a** Preoperative lateral X-ray films and CT three-dimensional (3D) reconstruction; (**b**) Diagram of hook plate fixation; (**c**) Posteroanterior and lateral X-ray films after 3 months postoperatively

**Fig. 4:**
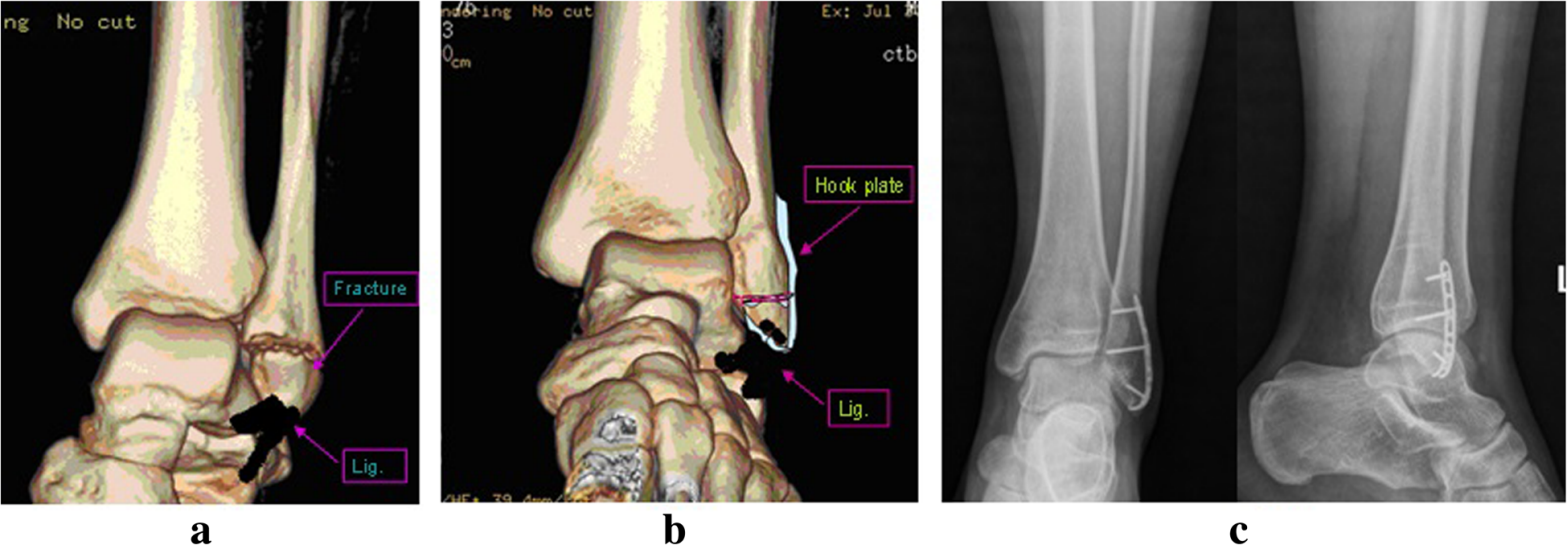
The radiographs of patients with lateral malleolus avulsion fracture treated with hook plate fixation. **a** Preoperative CT 3D reconstruction; (**b**) Diagram of hook plate fixation; (**c**) Posteroanterior and lateral X-ray films after 3 months postoperatively

The operation was performed in all patients under general anesthesia. The hook plates used in this study were AO hook plates (*n* = 21) or 1/3 titanium plates (*n* = 39) with two symmetric sharp teeth hooks, as shown in Fig. 1. After the removal of soft tissues at broken ends, bones were provisionally fixed with Kirschner wires prior to placing hook plates. Subsequently, hook plates were reconstructed to anastomose the bone surface according to inserted location and anatomical features of bones. The well-reconstructed hook plates were placed on the bone surface with sharp teeth-hooks fixed in the middle of the attachment points of ligament or tendon. Afterward, the hook surface was gently clicked to stick to fracture fragments. The Interventional C-Arm X-Ray imaging was used to confirm whether the bones were satisfactorily recovered and the hook plates were well-located. A cortical screw was passed through from the hole of hook plates for compressive fixation, while another cortical screw was inserted to maintain the reduction. In addition, the other cortical screw could be used to fix the greater fracture fragments by passing through from the crotch or lateral of the two hooks. As shown in Figs. 2, 3 and 4, the fragments of avulsion fractures were fixed by a hook plate or/and a screw. After the stability of remaining fracture fragments was maintained during continuous passive motion, negative pressure drainage was put and the skin was sutured.

### Postoperative care and functional evaluation

The limbs were treated with the assistance of continuous pressure by elastic bandages within 2 days post operation. Functional rehabilitation training began after anesthesia failure. One week postoperatively, patients with avulsion fractures of lower limb ligaments were encouraged to do some gentle exercise in the case of wearing the orthosis. From the 8th week, a free motion and weight bearing were allowed without orthosis. However, on the third day after surgery, patients with avulsion fractures of upper limbs were allowed to do recovery exercise without orthosis. All of the patients were reviewed and examined monthly after the operation and every 2–3 months after fracture union. One year later, the review period was changed to every 6 months. At the final follow-up, the functional recovery was assessed using the Lysholm knee scores [14], Kaikkonen ankle injury scores [15], Mayo elbow and wrist function scores [16], and Neer shoulder function scores [17].

### Statistical analysis

Statistical significance levels were determined by two-tailed t test or one-way analysis of variance (ANOVA). The *p* values of < 0.05 were considered significant. Data were presented as mean ± standard deviation (SD), and were analyzed using SPSS software (version 20.0; Chicago, IL, USA).

## Results

**Fig. 5:**
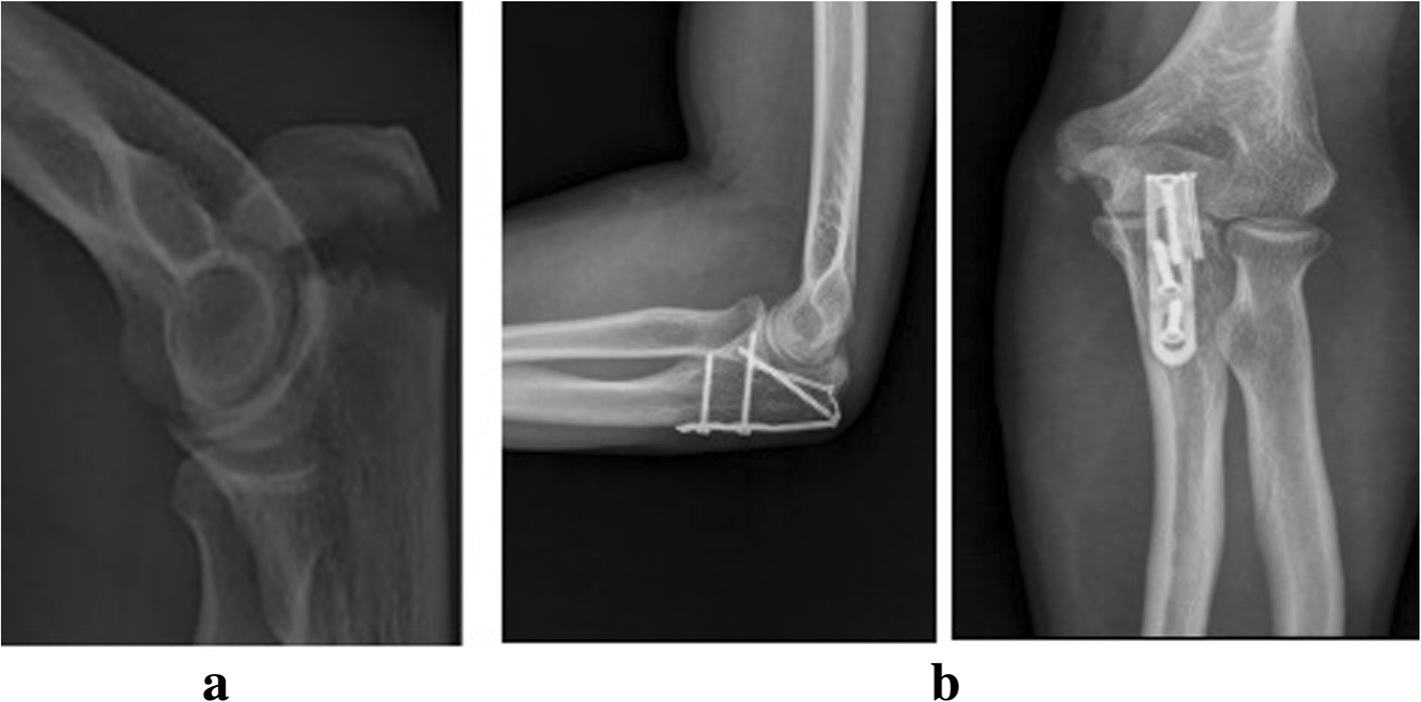
The radiographs of patients with left olecranon avulsion fracture treated with hook plate fixation. **a** Preoperative lateral X-ray films; **b** Posteroanterior and lateral X-ray films after 3 months postoperatively

**Fig. 6:**
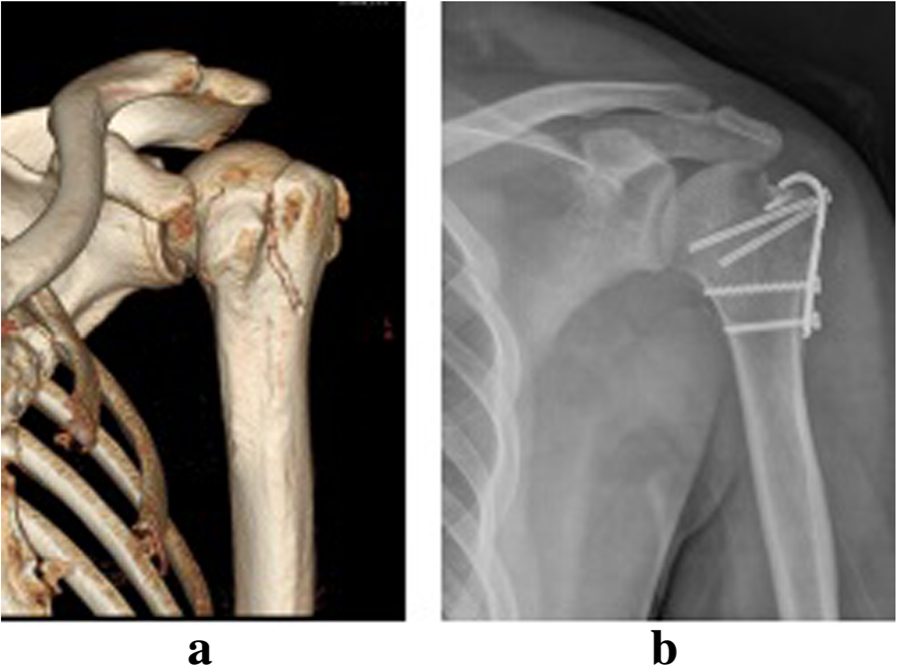
The radiographs of patients with avulsion fractures of greater tuberosity of humerus treated with hook plate fixation. **a** Preoperative posteroanterior X-ray films; (**b**) Posteroanterior X-ray films after 3 months postoperatively

All the patients were healed within 3 months after surgery with stage I healing incision without vascular or nerve injuries. The mean duration of follow-up was 18.1 months (range from 12 to 24 months). The functional score of the affected limb was significantly improved 3 months after surgery (Table 2, *p* < 0.05). No fractures with dislocation or delayed union were found in all patients. In the last follow-up, 3 cases (average age 55) with avulsion fractures of the humeral greater tuberosity had shoulder joint adhesion and peri humeral inflammation, and the functional recovery of shoulder joint was poor. Other patients were scored excellent or good of the functional recovery results. The total excellent and good rate was 95%. In addition, no instability of joints, looseness of internal fixation or traumatic arthritis was observed. Mild joint fibrosis occurred in 5 cases. In the last follow-ups, the functional score of the affected limb was markedly greater than that in the 3-month follow-ups (Table 2, *p* < 0.05). Internal fixation was removed after one year of surgery in 35 patients. The follow-up data for functional scores at each site was shown in Table 2. Typical cases were presented in Figs. 2, 3, 4, 5 and 6.

**Table 2:** The follow-up data for functional scores at each site

Fracture site	Case	Functional score		
		Preoperation	3 months post operation	Last follow-ups
Greater tuberosity of humerus	8	42.0 ± 2.1	67.9 ± 5.5*	84.8 ± 6.3^#^
Medial epicondyle of humerus	6	50.5 ± 1.9	73.8 ± 2.9*	92.3 ± 1.6^#^
Ulna olecranon	17	48.4 ± 1.9	70.5 ± 2.9*	90.7 ± 1.6^#^
Ulnar styloid	4	55.5 ± 2.1	79.0 ± 1.0*	91.5 ± 1.3^#^
Lateral malleolus of tibia	6	50.5 ± 2.1	72.5 ± 1.9*	92.0 ± 1.4^#^
Knee PCL	19	50.2 ± 1.4	70.0 ± 1.7*	91.4 ± 1.9^#^

*p < 0.05 vs. Preoperation; #*p* < 0.05 vs. 3 months post operation. *PCL*: posterior cruciate ligament

## Discussion

Avulsion fracture around joints is a form of ligament or tendon injury, and is also a special type of intra-articular fracture [18, 19]. On the one hand, the traction of ligaments, tendons, or articular capsules increased the occurrence of displacement. On the other hand, soft tissues are often embedded in the broken end. These two points make the fracture reduction become difficult, increasing the incidence of nonunion and affecting the functional recovery of ligaments, tendons and joints [12, 13]. Additionally, patients with defects of articular cartilage or articular surface would suffer from arthritis or joint dysfunction. Commonly, conservative treatment and surgery are the two major treatments of avulsion fracture. However, conservative treatment often causes some complications, such as synarthrophysis, nonunion, joint instability, and arthritis, resulting in an unstable fixation and an unsatisfactory outcome. Therefore, most avulsion fracture is recommended to be treated with internal fixation in the early stage. Indeed, patients with avulsion fractures without displacement was suggested to receive surgical treatment to promote the functional recovery [13, 14]. For instance, Huang et al. [19] found that the anterior arthroscopic-assisted fixation was a simple and feasible alternative for treating PCL avulsion fractures.

At present, the commonly used internal fixation methods for avulsion fracture include screws, steel wires, K-wire tension band, suture anchors, sutures, straddle nails, and allogeneic bone nails, among which screw fixation is the most commonly used. The stability of screw fixation is reliable for larger avulsion fracture fragments, but for patients with osteoporosis, small fracture fragments, or severe comminution, it is easy to damage the bone fragments when drilling or screwing. Therefore, screw fixation is more suitable for avulsion fracture patients with large bone mass and without heavy osteoporosis [18, 20]. For comminuted fractures that cannot be fixed with screw, steel wires, K-wire tension band, and sutures can be selected. However, these operations need to be performed in the transitional zone between the fragment and ligament, which is inconvenient and relatively cumbersome. When fixing with fine stainless-steel wire, excessive tension will injure ligaments and damage fracture fragments.

The hook plate used in surgical treatment of avulsion fractures had several advantages as follows [1, 13, 21] It was established from AO special hook plate or 1/3 titanium plate, which was commonly used in fracture treatments. (2) The hook plates established a non-rigid fixation to maintain stability, allowing a longer period for retention of the implant. (3) Eccentric screws were implanted independent of fracture fragments through the hook plates for compress fixation. (4) The hook plates declined boring and cutting damages of fracture fragments in favor of bone union. (5) The hook plates were performed regardless of the size of ligaments, tendons and fragments. (6) It had wide indications, particularly for hand avulsion fracture. In addition, Shin et al. [12] compared the mechanical effects of the hook plate and the rivet fixation in the treatment of ulnar collateral ligament avulsion fracture of metacarpophalangeal joint, and the results showed that the stability and strength of the hook plate were better than the rivet. Our previous research compared hook plate with traditional method in the treatment of avulsion fracture of the olecranon, and found that all fractures healed. There was no significant difference in healing time and incidence of complications between the two groups (*P* > 0.05). However, the former was superior to the control group in terms of functional recovery and range of joint activity, and the difference was statistically significant (*P* < 0.05) [22].

In this study, 3 cases with avulsion fractures of greater tuberosity of humerus had shoulder joint adhesion and perihumeral inflammation, and the functional recovery of shoulder was poor. One reason is due to the high age of the patients (average age was 55). In addition, the patients did not actively cooperate with the treatment for early rehabilitation exercises because of their low requirements for shoulder joint function after the operation. Meanwhile, the hook is located above the greater tubercle, which may impact the acromion and affect shoulder joint activity. Therefore, anchor suture and fixation should be the first choice for avulsion fracture of greater tubercle of humerus. Our study was designed to evaluate the outcomes of the hook plate fixation for 60 patients with avulsion fractures around joints. All the patients performed early rehabilitation exercises and the incision healed well without complications, such as screw loosening, instability of joints and arthritis. The excellent and good rate of joint function recovery was about 95%, which was higher than traditional internal fixation [12]. Nevertheless, hook plate fixation still had some limitations. Firstly, hook plate fixation cannot be used under arthroscope. Secondly, it was unsuitable for severe comminuted fractures and anterior cruciate ligament avulsion fractures. Consequently, we should choose the appropriate internal fixation method according to different clinical situations.

In conclusion, hook plate fixation has the therapeutic effect on treating avulsion fractures around joints with the advantages of reliable fixation, early rehabilitation after operation, high recovery rates of joint function, wide indications, and convenient uses.

## Abbreviations

CT: Computed tomography
MRI: Magnetic resonance imaging
PCL: posterior cruciate ligament
UCL: ulnar collateral ligament
